# Dame Swift on Nursing Abuses

**Published:** 1920-09-25

**Authors:** 


### DAME SWIFT ON NURSING ABUSES.

The correspondence on nurses' legitimate grievances to which the *Globe* has opened its columns contains matter of very unequal value. Some of the writers are frankly more than a little rancorous. But hard words break no bones. They merely accentuate the gulf between the professional outlook and that of outsiders. Into the turbid waters of controversy the good sense and sound information of Dame Swift intervene with marked effect. It has been her part to testify to the enormous increase of late years in the nurse's work and responsibilities owing to advances in medical science, and in her judgment this increase has not been followed by a commensurate increase in salaries. It has, in fact, met with little recognition, owing probably to the very gradual nature of the processes which have brought it about. Dame Swift considers that the claims of nurses have been largely overlooked, that the standard of education required for a fully-trained nurse or hospital sister is now very high, and that the remuneration has not been raised to meet the increased cost of living. A nurse paid at the pre-war rate earns barely enough to provide her with boots and shoes and other articles of clothing. "Women in other professions, which require the same or a lower standard of education, are earning three or four times the amount of the trained nurse's salary."

In reply to the query, "How much should a nurse be paid?" Dame Swift asserts that a fully certificated nurse ought to be entitled to a minimum salary of £70 a year, in addition to board, lodging, and uniform, while a sister ought to receive anything up to £300 a year, according to the size of the institution and the responsibilities of the charge. Dame Swift is in touch with hospitals all over the kingdom, and announces that many have already fallen in with the recommendations of the College of Nursing. In fact, she was able to state that there is not a large hospital known to her in which an effort is not being made to provide an eight hours' day for the nurses, and that the College of Nursing has already done a great deal to secure for nurses improved conditions, with shorter hours and better pay. Having pointed out that reforms are being retarded for lack of accommodation, Dame Swift gave her opinion decidedly against the living-out system, because it would make it impossible to ensure that the nurse had either sufficient food or rest.

So clear and candid an examination into the causes of nurses' difficulties cannot fail to be productive of much good.

